# Serum resistin is associated with C-reactive protein and LDL- cholesterol in type 2 diabetes and coronary artery disease in a Saudi population

**DOI:** 10.1186/1475-2840-4-10

**Published:** 2005-07-05

**Authors:** N Al-Daghri, R Chetty, PG McTernan, K Al-Rubean, O Al-Attas, AF Jones, S Kumar

**Affiliations:** 1 King Saud University College of Science, Biochemistry Department, Riyadh, Saudi Arabia; 2 Birmingham Heartland Hospital, Clinical Biochemistry, Birmingham B9 5SS, UK; 3 King Saud University, College of Medicine, Medicine Department, Riyadh, Saudi Arabia; 4 University of Warwick, Warwick Medical School, Diabetes & Metabolism Unit, Coventry, CV4 7AL, UK

**Keywords:** type 2 diabetes, Coronary Artery disease, resistin, C-reactive protein

## Abstract

**Aims:**

Resistin is an adipocyte-derived factor implicated in obesity-associated type 2 diabetes (T2DM). This study examines the association between human serum resistin, T2DM and coronary heart disease.

**Methods:**

One hundred and fourteen Saudi Arabian patients (male: female ratio 46:68; age 51.4 (mean ± SD)11.7 years; median and range: 45.59 (11.7) years and BMI: 27.1 (mean ± SD) 8.1 Kgm^2 ^median and range: 30.3 (6.3) were studied. Serum resistin and C-reactive protein (CRP), a marker of inflammation CRP levels, were measured in all subjects. (35 patients had type 2 diabetes mellitus (T2DM); 22 patients had coronary heart disease (CHD).

**Results:**

Serum resistin levels were 1.2-fold higher in type 2 diabetes and 1.3-fold higher in CHD than in controls (p = 0.01). In addition, CRP was significantly increased in both T2DM and CHD patients (p = 0.007 and p = 0.002 respectively). The use of regression analysis also determined that serum resistin correlated with CRP levels (p = 0.04, R^2 ^0.045).

**Conclusion:**

The findings from this study further implicate resistin as a circulating protein associated with T2DM and CHD. In addition this study also demonstrates an association between resistin and CRP, a marker of inflammation in type 2 diabetic patients.

## Introduction

Chronic sub-clinical inflammation has been identified as an important mechanism in the pathogenesis of T2DM and associated cardiovascular complications [1]. Obesity, a major risk factor for T2DM and cardiovascular disease, is now also established as a state of chronic inflammation [2]. For this reason, adipose tissue may represent an important site for the production of pro-inflammatory cytokines and acute phase reactants [3]. Such pro-inflammatory factors include tumour necrosis factor alpha (TNF-α), interleukin 6 (IL-6) and leptin, which are secreted by adipocytes [4-7] and are elevated in obesity and insulin resistant states. This may therefore suggest the adipocyte as a site also for an initial inflammatory response, therefore increasing the overlap between metabolic and inflammatory signalling pathways.

The adipocytokine resistin which belongs to a family of cysteine-rich C-terminal proteins known as resistin-like molecules (RELM; RELMα/FIZZ 1 and RELMβ/FIZZ 2) of FIZZ (found in inflammatory zone) are thought to be involved in inflammatory processes [8-11]. Previous studies have however highlighted that in mice, resistin impairs glucose tolerance and insulin action. In addition, resistin also inhibits adipogenesis in murine 3T3-L1 cells [8,11-14]. Therefore resistin has also been proposed as an adipocyte-secreted factor thought to link obesity and T2DM [9], although subsequent rodent studies have reported contrasting findings to this [15]. In addition, the role of resistin in human obesity has also produced conflicting reports, and at present resistin still remains controversial as a potential mediator in the pathogenesis of T2DM and the impact of resistin on T2DM status [16-21].

Our previous studies have highlighted that serum resistin levels are increased in Caucasian T2DM subjects and reduced with modest weight loss [18,22] and these factors are also associated with changes in C-reactive protein (CRP) levels, CRP represents one of the acute phase proteins which increase during systemic inflammation. CRP is a known inflammatory marker for T2DM and coronary heart disease (CHD) [23]. Our previous cohort studies have also determined that with T2DM patients CRP represents an independent predictor of serum resistin levels [18]. Furthermore, it is apparent that ethnicity can affect the relative risk of T2DM and cardiovascular complications with South Asians, Arabs and Afro-Asians, with T2DM having a higher adverse risk profile [24-29]. However, to date the relationship between serum resistin levels, and T2DM or CHD has not been investigated in a Saudi Arabian population. Therefore the aims of this study were to investigate for the first time whether serum resistin levels are increased in Saudi Arabians with T2DM and if this is affected by CHD. Furthermore these studies seek to ascertain whether CRP is correlated with serum resistin levels in a Saudi Arabian population.

## Materials and methods

### Subjects

The type 2 diabetic patients for this study were randomly selected from the Diabetes Center and non-diabetic case control patients were obtained from out-patients of King Abdulaziz University Hospital at King Saud University in Riyadh, Saudi Arabia. Coronary heart disease (CHD) patients were selected from the Coronary Care units at King Khalid University Hospital and Prince Sultan Cardiac Units in Riyadh. From these, 114 Saudi Arabian patients were studied (Table 1). CHD patients (n = 22) were classified as having coronary heart disease (CHD) if they had suffered any of the following: a myocardial infarction, angina with a positive exercise tolerance test, a resting ECG consistent with CHD or angiographically proven coronary artery disease. T2DM patients (n = 35), diagnosed according to WHO criteria, but with no clinical evidence of CHD were screened with ECG, clinical symptoms and ± thallium cardiac scanning or cathertization. All patients with T2DM were treated by diet with or without oral hypoglycemic agents, mainly glibenclamide and metformin. Finally 50 Saudi individuals with no prior history of CHD or T2DM were recruited as healthy case controls from general practice. Ethical approval was obtained from the local institution's review committee.

**Table 1:** Clinical Characteristics of all patients

	**Case Control**	**T2DM**	**CHD**
**N**	50	35	22
**Age (years)**	42.5(10.0)	53.6(11.7)^a^	47.6(11.9)
**Diabetes duration (years)**	0	6.8(6.3)	0
**Systolic BP (mm/Hg)**	117.7(16.7)	124.1(25.2)	135.0(20.7)
**Diastolic BP (mm/Hg)**	76.8(11.6)	78.8(14.5)	78.8(14.6)
**BMI (KG/M^2^)**	29.7(6.3)	27.5(6.9)	33.7(15.9)
**Waist (cm)**	94.1(15.0)	92.7(9.5)	91.4(12.2)
**Hips (cm)**	105.7(17.1)	95.0(15.6)	98.6(12.2)

Data are presented as mean ± (SD). Clinical characteristics for case control versus either type 2 diabetic or CHD patients were assessed (p-value: a = p < 0.01).

All patients underwent a full physical examination and completed a general questionnaire. Body mass index (BMI), blood pressure, smoking habits and age were recorded. Blood samples were collected after a 12-hr fast for the determination of total cholesterol, HDL, triglycerides, insulin, apolipoprotein AI (Apo AI) and apolipoprotein AII (Apo AII), leptin and serum resistin concentrations. These studies were conducted with the approval of the King Abdulaziz Diabetic Center with approval obtained from the ethics committee and the individual consent of each patient.

### Assays

Plasma samples were stored at -70°C prior to analysis. Measurements of serum cholesterol, HDL, and triglycerides, Apo AI and ApoAII were performed using routine laboratory methods. Insulin was analysed by a solid phase enzyme amplified sensitivity immunoassay (Medgenix INS-ELISA, Biosource, Belgium). Insulin resistance (HOMA IR) and β-cell function index were derived using the HOMA equation [27]. Leptin concentrations were measured by radio-immunoassay (Linco Research, St. Charles, MO). CRP was analysed by immunoturbidimetric method (Roche/ Hitachi analyser). Serum resistin was measured using the human resistin ELISA assay from Phoenix Pharmaceuticals (Belmont, California, USA; intra-assay variability <5%). The commercially available resistin ELISA had been validated as part of previous studies [15].

### Statistical Analysis

Data were stored and analysed using the SPSS version 10 package (SPSS, Evanston, IL, USA) for Windows. Biochemical parameters not normally distributed were analysed after being logarithmically transformed. A student's unpaired t-test or one-way ANOVA exposed the differences between the groups. Simple and partial correlation coefficients between the variables were determined and multiple regression analysis was performed to determine the relationships between the variables of interest. Data were expressed as mean and standard deviation (SD) or median and range; statistical significance was accepted at p < 0.05.

## Results

### Clinical characteristics

The full clinical and biochemical characteristics for the three groups are set out in Tables 1 and 2 respectively. The control subjects were noted to be younger than the T2DM patient counterparts (p < 0.01). However, there were no significant differences in age or blood pressure, Body mass index (BMI) or waist circumstance between case controls and CHD patients.

### Metabolic Characteristics

**Figure 1:**
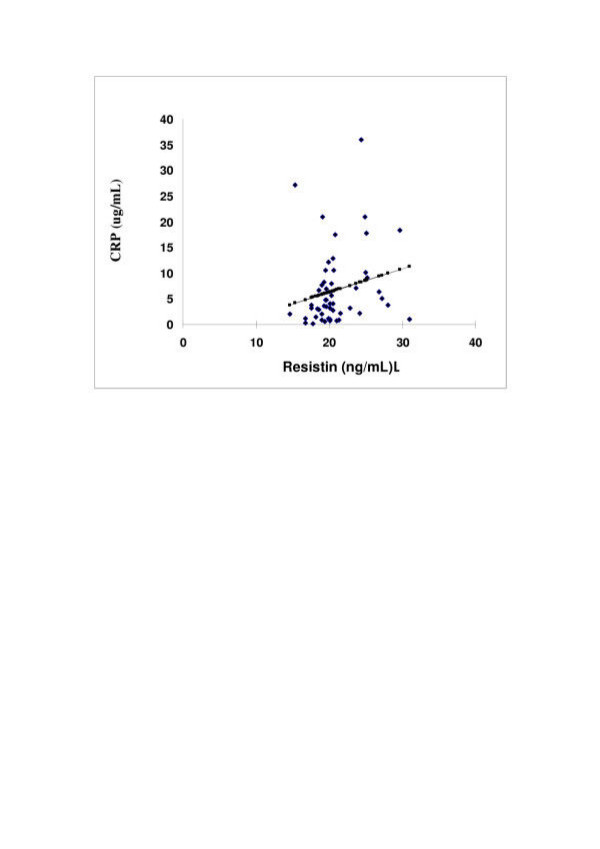
The correlation of Serum resistin levels (ng/mL) with C-reactive protein (mg/mL) using univariate analysis across all Saudi subjects, case controls, T2DM and CHD subjects. Black denotes the actual values with the line of best fit shown in square (R^2 ^0.045 p-value = 0.04).

Serum resistin was significantly higher in T2DM 22.6(± 4.5)ng/mL and CHD patients 24.2(± 3.6) ng/mL compared with case controls 18.9(± 3.4) ng/mL (p-value <0.0001). CRP was significantly increased in T2DM 8.2 (0.7–27.1) μg/mL median (range) and CHD patients 7.3 (1.8–18.3) μg/mL(p = 0.007 and p = 0.002 respectively) compared with case controls 4.9(0.2–18.9) μg/mL (Table 2). Regression analysis determined that serum resistin was associated with serum CRP levels (Figure 1).

**Table 2:** Metabolic Characteristics of all patients

	**Case Control**	**T2DM**	**CHD**
**N**	50	35	22
**Fasting Glucose mmol/L**	5.4(1.4)	15.4(5.4)^a^	5.6(0.6)
**Cholesterol mmol/L**	5.3(1.3)	6.9(1.9)^b^	7.7(2.7)^c^
**HDL mmol/L**	0.9(0.4)	0.88(0.3)	0.92(0.4)
**Triglyceride mmol/L**	1.8(0.6)	2.5(1.1)	2.1(1.2)
**LDL mmol/L**	3.9(1.4)	6.1(2.9)^d^	4.9(1.9)^e^
Insulin (Range) μmol/L*	7.5(5–12)	9.0(9–16)	7.0(1–49)
Leptin (Range)ng/mL*	7.2(2–30)	5.8(1.9–28)	3.7(0.7–8.9)
**Apo1 mg/dL**	80.1(15.6)	70.8.(23.4)^c^	74.1(24.5)
**Apo11 mg/dL**	48.7(13.5)	45.9(13.6)^f^	46.3(18.9)^c^
HOMAIR (Range)*	1.8(1.3–3.2)	8.2(0.8–49.7)	2.0(0.5–5.6)
**Resistin ng/mL**	18.9(3.4)	22.6(4.5)^e^	24.2(3.2)^d^
CRP (Range) μg/mL*	4.9(0.2–18.9)	8.2(0.7–27.1)^g^	7.3(1.8–18.3)^f^

Data are presented as means (SD) or as median (interquartile range) for insulin, leptin HOMIR and CRP data. P-values shown are comparison of metabolic characteristics for case control versus either type 2 diabetic (T2DM) or coronary heart disease (CHD) patients as highlighted: a = p < 0.0001, b = p < 0.05, c = p < 0.003, d = p < 0.017, e = p < 0.01, f = p < 0.002, g = p < 0.007.

Fasting plasma glucose 15.4(5.4) serum cholesterol 15.4(5.4) mmol/L, LDL6.1(2.9) mmol/L and Apo-AI 70.8 (23.4)mg/dL were higher in T2DM patients (p < 0.0001, p < 0.05, p < 0.017, p < 0.003, respectively) compared with case control subjects, while the Apo-AII 45.9 (13.6)mg/dL was lower in the T2DM than in the controls, where p < 0.002 (Table 2). In CHD, cholesterol 7.7 (2.7), and LDL 4.9(1.9) mmol/L were significantly higher than in the case controls (p = 0.003, p < 0.01 respectively; while the Apo-AII 46.3(18.9) mg/dL in CHD patients as lower than in the case controls, where p = 0.003 (Table 2).

### Univariate analysis

**Figure 2:**
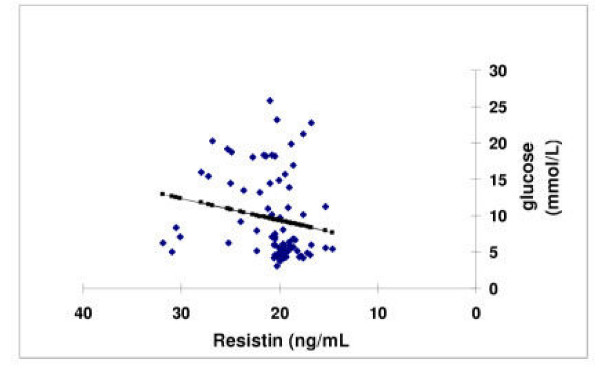
The correlation of Serum resistin levels (ng/mL) with glucose (mmol/L) across Saudi subjects with univariate analysis across all case controls, T2DM and CHD subjects. Blue denotes the actual values with the line of best fit shown in pink (R^2 ^= 0.03, p-value = 0.01).

Univariate analysis for all groups showed strong positive associations between resistin and fasting glucose (p = 0.01, Figure 2), and negative association with hip circumference (R^2 ^0.6, p = 0.004, p = 0.03 respectively) but in CHD patients, resistin had a strong positive association with systolic and diastolic blood pressure and Apo-AII (R^2 ^0.82, p < 0.0017, R^2 ^= 0.45 p < 0.002 respectively). There was also a strong correlation with Apo-AII (p < 0.003), whereas, in T2DM patients, resistin was correlated with LDL (R^2 ^= 0.79, p = 0.009).

## Discussion

While it is apparent there are conflicting reports as to the role of serum resistin and its role in obesity mediated T2DM, no study to date has examined this in relation to a Saudi population with and without T2DM and/or CHD. This study determined that serum resistin levels were increased in T2DM and CHD in a Saudi population, when compared with case control subjects.

In addition it was demonstrated that a correlation exists between serum resistin and CRP levels in Saudi subjects, confirming our studies in Caucasian non-diabetic and T2DM subjects [18]. These data further suggest a potential role for resistin as a marker associated with inflammation in both T2DM and CHD disease. Studies have already shown pro-inflammatory cytokines to be important inducers of CRP, such as IL-6, TNF-α, and leptin [31], and resistin may also play a role as an inducer of CRP.

Other studies have examined the relationship between BMI and serum resistin levels. However, while some studies have shown such results as correlation [16], this study and our previous study [18] failed to determine such an effect. The relationship of resistin with increasing adiposity was not a primary determinant of this study and as such the range of BMI across this cohort may not be sufficient to identify a correlation. However, a negative correlation of serum resistin with hip circumference was noted, supporting a possible link between resistin and obesity.

Our earlier and present studies also investigated whether there was any correlation between serum resistin levels and fasted glucose levels across all the subjects. A significant correlation between fasting glucose and serum resistin was observed. This supports the previous finding by Lazar and his co-workers from their rodent model studies, where fasted blood glucose was higher in resistin-transgenic mice than in their non-transgenic littermates, and glucose tolerance was impaired in the hyper-resistinemic mice [32].

However *in vivo *human analysis of the relationship between serum resistin and glucose has produced conflicting reports [17,18,33,34]. No correlations were identified in serum resistin and glucose amongst non-obese, obese and obese T2DM subjects by examining the glucose disposal rate during a hyperinsulinaemic glucose clamp across groups [17]. In conjunction with this, an *in vivo *analysis by Azuma *et al. *also noted no significant correlation in serum resistin and glucose with cross-sectional analysis; but longitudinal analysis in the same cohort revealed changes in serum resistin to be positively correlated with glucose as well as other factors such as BMI, and insulin [34]. Hence, these studies may suggest that cross sectional analysis of serum resistin may also be inadequate to determine correlations. Further, the specificity of the resistin ELISA assay emerges as an important factor in these and other studies; to date, however, only one commercial ELISA appears to have been validated for cross-reactivity with different members of the RELM family and this may affect the findings in other studies [18].

Additional *in vitro *studies in human adipose cells supports the role of resistin in reducing glucose uptake, with the effect of revealing a potential functional role for resistin in *in vivo *metabolism [18].

In conclusion, these present findings suggest that resistin may have a dual role involved in sub-clinical inflammation as well as in altering glucose metabolism leading to the progression of T2DM in this Saudi cohort. As such, this study may add to the current speculation over the roles and actions of resistin, since studies continue to suggest a role for resistin in affecting glucose, insulin sensitivity and sub-clinical inflammation.
